# Fabrication of benzoyl chloride treated tiger-nut fiber reinforced insect repellent hybrid composite

**DOI:** 10.1038/s41598-022-12876-0

**Published:** 2022-05-25

**Authors:** Hajara Babayo, Haruna Musa, Mustapha D. Garba

**Affiliations:** 1 Department of Pure and Industrial Chemistry, Bayero University Kano, P.M.B 3011, Kano, Nigeria; 2 Fibre and Polymer Science Program, North Carolina State University Raleigh, Raleigh, NC 27606 USA; 3 School of Chemistry, University of Glasgow, Glasgow, G12 8QQ Scotland, UK

**Keywords:** Mechanical properties, Materials for devices

## Abstract

An insect repellent composite containing tiger nut particulate fibre, waste low density polyethene (LDPE) and castor oil alkyd resin was fabricated. *Canarium schweinfurthii* gum was used as insect repellent compound and maleic anhydride as compatibilizer. The tiger nut chaff was subjected to benzoylation using benzoyl chloride to increase the fibre-matrix interaction. The compound and composition was then moulded with LDPE as dual matrices for excellent physico-mechanical properties (pressed for 5 min, 130 °C and 25 bar and cured). The 10 wt.% treated composite exhibited a minimum water absorption of ~ 0.085%, optimal chemical resistance for both acids (HCl and H_2_SO_4_) and bases (NaOH and KOH) and no effect on thickness. Density measurement showed the lowest value of ~ 0.0096 g/cm^3^ for the treated fibre composite. However, the tensile strength, flexural stress, hardness and impact load were improved up to 35.08 Mpa, 456.3 Mpa, 95 and 730 J/m respectively with treated composites. Insect repellent tests against termites and cockroaches show repellent activity with time intervals. FTIR and SEM analysis showed fibre modification achieved.

## Introduction

Tiger nut has been reported to contain high fibre, with lots of other natural nutrients. It is processed as a juice drink in Africa, especially in Nigeria, with scant scientific attraction^1,2^. The chaff is usually treated as waste, but in some instances people use it to feed domestic animals. However, it can be utilized in composite fabrication due its fibre content.

The Canarium schweinfurthii Engl. trees are found in Central, East, and West Africa commonly used in traditional medicines. The resin is usually used as mosquito repellent, while oil extract has been used to kill termites and has been found to be highly potent^3^.

LDPE is one of the predominant consumer petroleum-based synthetic plastic polymers difficult to decompose over decades, with ~ 70% increase waste production projected globally^4^. Nigeria is one of the countries that uses LDPE for packaging with no sustainability consideration. This can be utilized for composite fabrication due to excellent chemical resistance, high impact strength, flexibility and hydrophobicity^5^.

Composites are developed using combination of natural and synthetic polymers with many already used for industrial applications^6^, with synthetic polymers such as polystyrene (PS), polypropylene (PP), polyvinyl chloride (PVC) and polyethylene (PE) extensively studied^7^. Literature shows that polyethylene has good properties (such as low density, hardness, flex life, scratch resistance and is relatively low cost) suitable for composites production^8^. Reinforcement of such a composite can be achieved with natural plant fibres in combination with a non-biodegradable polymer matrix such as, polyethylene^9^. The survival of natural fibre for this purpose depends on the fibre treatment. Although synthetic polymers are known to have improved mechanical properties when compared with natural polymers, they are consequently environmental pollutant and non-degradable^7,10^.

Composites are a combination of materials consisting of reinforcing phase (i.e. particles sheet and/or fibers), embedded in a matrix phase, where both materials can be polymer, metal, or ceramic^11,12^. Hybrid composites are regarded as more advanced in comparison to the conventional fibre-reinforced polymer (FRP) composites^13,14^. The fibres usually offer unidirectional reinforcement, while addition of polymers increases stiffness, thermal expansion coefficient, and results in high permeation resistance of liquids and gases^15^. To increase the thermal and mechanical properties of prepared composites, chemical treatments are used to expose the fiber reactive group for efficient matrix coupling. This is done via mercerization, silane and/or benzoylation that involve simple immersion of the fiber into the chemical solution at ambient temperature^16^.

Benzoylation chemical treatment using Benzoyl chloride (C_6_H_5_ClO) is mostly commonly used for treatment of composites, thereby decreasing the hydrophilicity and improving the fibre matrix adhesion (enhanced thermal stability and strengthen fiber)^17–19^. Alkali pre-treated fibre provides even higher thermal stability^19^. Work by Wang et al.^20^ involving similar chemical treatment reports 6 and 33% tensile strength and moisture resistance improvement respectively.

This work intends to explore the use of environmentally friendly material (plant resin/extract) with insect repellent property to formulate a natural fibre reinforced composite with excellent mechanical and termite repellent properties. The research looked at the concept of "waste to wealth" by converting the waste LDPE and tiger nut chaff to composite, with potential application in construction and furniture having repellent activity using natural Canarium sweinfurtii plant exudate. In this paper, the insect-repellent hybrid composite with improved mechanical, chemical and physical properties was successfully prepared. It has potential in the production of ceiling panels and furniture making to repel domestic destructive insects such as termites and cockroaches, which is significant for the development of sustainable and environmentally acceptable producing technology.

## Materials and experimental methods

### Material

Castor oil purchased from NARICT Zaria, Nigeria. Sodium hydroxide, NaCl, sulfuric acid, ethanol, acetone, xylene, toluene, and cyclohexanol with > 97% purity were purchased from Sigma-Aldrich. Glycerol, maleic anhydride and phthalic anhydride with > 95% purity were purchased from Emerch India. *Canarium* schweinfurthii (insect repellent) was obtained locally in Nigeria. The plant collection is in compliance with relevant guidelines approved by the Directorate of Research, Innovation and Partnership (DRIP), Bayero University, Kano. Tiger-nut seed, purchased from a local market. LDPE; waste sachet water bags were collected from local shops at Bayero University Kano State in Nigeria.

### Experimental procedures

#### Preparation of free fatty acid alkyd resin

The alkyd resin was prepared from castor oil. The crude castor oil was first purified by vacuum filtration according to the method described by Feist and Fessenchen^21^. The physicochemical properties, methods, as well as the instruments used are shown in supplementary table S1. The analyses were performed to ascertain the quality of the crude castor oil. All physicochemical properties obtained fall within the standard ranges.

**Figure 1:**
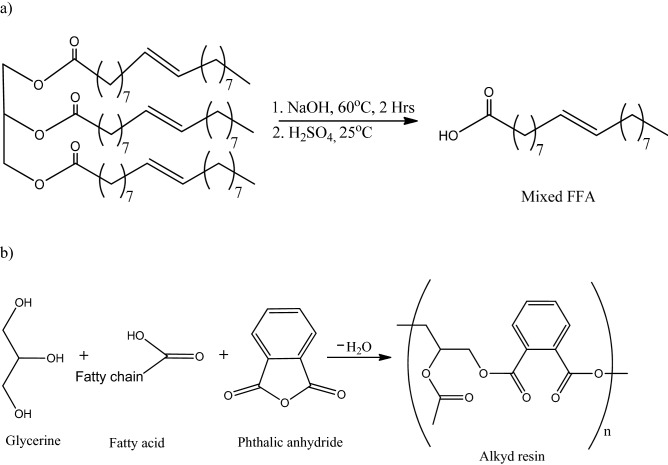
(**a**) Extraction of FFA by saponification and acidification of crude castor oil. (**b**) General reaction for fatty acid alkyd resin.

After the analysis of the crude castor oil, extraction of the free fatty acid was performed using a method described by Bat^22^ with little modification. NaOH (25 g) was dissolved in 50 cm^3^ (1:1) of ethanol-distilled water to give a solution which was used to saponify the castor oil (100 g). The mixture was left to react under reflux at 60 °C for 2 h until a homogeneous mixture was obtained (set up included: dean and stark, hot plate, reflux condenser and three necked round bottom flask). The next step was phase separation, which was accomplished by using 100 cm^3^ of 6.15 M NaCl solution (saturated solution). The soap produced is collected at the upper layer, while the glycerol goes to an aqueous phase in the lower layer. The soap was then filtered out by means of a vacuum filtration to remove the aqueous phase (supplementary Fig. S1). The residue is dissolved in 100 cm^3^ distilled water and then reacted with a stoichiometric amount of sulfuric acid (34 g) at room temperature (scheme in Fig. 1a). The fatty acid extracted was collected from the upper layer and then separated using a separating funnel and further centrifuged at 5000 rpm for 10 min to get rid of any remaining water. Finally, the unsaturated fatty acid was dried with anhydrous Na_2_SO_4_ and characterized. Castor oil alkyd resin was synthesised by reaction of FFA, glycerol and phthalic anhydride according to the modified method reported by Keskin^23^ as presented in scheme Fig. 1b.

The extracted free fatty acid (55.14 g, 62.27 wt.%), glycerol (13.72 g, 15.10 wt.%), phthalic anhydride (19.68 g, 22.23 wt.%), ratio 8:2:3 was fed into round bottom flask (a three-necked) fitted with dean and stark apparatus, a thermometer and placed on a heating mantle (supplementary Fig. S2). Xylene (10.62 g or 12.2 cm^3^, 6% of the total weight of feed) and N_2_ gas (30 ml/min) were both used to remove reaction water from the esterification reaction. The reaction was performed at 170 °C, followed by acid number monitoring. As the reaction proceeds, the acid number decreases regularly and the reaction stopped at 30 mg KOH/g. The N_2_ purging rate slightly increased (35 ml/min) to remove the liberated reaction product and to increase the heat mass transfer of the chemical reaction. The Alkyd resin with acid number 25 mg KOH/g was obtained after 4 h, 10 min. As the acid value of the resin decreases the viscosity increases, hence the reaction should not proceed after the acid number dropped to 25 mg KOH/g to avoid gelation and viscosity build up^24^.

The solubility of the alkyd resin sample in different solvents, such as acetone, ethanol, xylene, toluene, and cyclohexanol was evaluated. The resin (1 g) was weighed into 5 different beakers; 10 cm^3^ of each solvent, were added individually, shaken vigorously and the solubility was carefully observed and recorded (supplementary table S6).

#### Preparation of the composite

The composite fabrication comprises of insect-repellent binder (*Canarium* schweinfurthii), reinforcement (tiger-nut seed), hybrid matrices (LDPE; waste sachet water bags and castor oil free fatty acid Alkyd resin) and compatibilizer (maleic anhydride) (Supplementary Fig. S3).

Benzoylation was carried out on the tiger nut particulate fibre by modification of a method reported by Bassyouni and Hasan^25^. Un-treated tiger nut fibre (100 g) was soaked in 300 cm^3^ NaOH (18% w/v) solution for 30 min, filtered and washed with distilled water. The pre-treated fibres were suspended in a 100 cm^3^ NaOH (10%) solution and agitated with 50 cm^3^ benzoyl chloride. The mixture was kept for 15 min, filtered, washed and dried. The fibres were then treated with ethanol for 1 h to remove the unreacted benzoyl chloride. Finally, the fibre washed with distilled water and dried overnight. Both the untreated and treated tiger nut particulate fibres were then characterised.

#### Moulding and pressing of the fabricated composite

The composites were fabricated using compression moulding techniques (Hydraulic hot press Carver Inc. model 3851–0). The waste LDPE and alkyd resin matrices were mixed using a two-roll mill (Reliable rubber and plastics machinery company, New Jersey, U.S.A, model 5189) in the ratio 100:5 parts by weight. *Canarium schweinfurthii* gum (30-60 g) which serves as a binder, treated/untreated tiger nut particulate fibre (10-30 g) and maleic anhydride compatibilizer (3 g) were also added as presented in Table 1. All the mixing was done using two mill rollers and the product was transferred to a 15 × 12 mm mould with thickness of 3 mm, pressed for 5 min at 130 °C, 25 bars and then cured as shown in supplementary Fig. 4.

**Table 1:** Formulations of composites fabrication with the particulate fibre.

Material	Part by hundreds of LDPE			
	Control	10% Fiber composition	20% Fiber composition	30% Fiber composition
*Canariun schweinfurthii* (Binder)	60	50	40	30
Tiger nut particulate fibre	_	10	20	30
Alkyd resin	5	5	5	5
Maleic anhydride	3	3	3	3

### Analytical methods

All the analyses were carried out according to standard methods. The various standard methods including the instrument specifications are shown in supplementary Table S10.

The water absorption test was performed using ASTM D-570 standard procedure; the test was carried out on both treated and untreated fibre composites. The experiment repeated 3 × and the average taken. Weighed Composite (w_1_) was immersed in distilled water for 24 h, re-weighed after immersion (w_2_), and the percentage gain/loss was calculated using the following equation^26^:

1 $$\left( {\% {\text{weight}}\;{\text{gain}}/{\text{loss}}} \right) \, = \, \left( {{\text{W2}} - {\text{W1}}} \right)/{\text{W1 }} \times {1}00\%$$

W_1_ = Initial weight of the composite sample. W_2_ = Final weight of the composite sample.

The thickness swelling analysis was performed using ASTM D 570 as reported by Salisu et al.^26^. The thickness measured and recorded before (T_1_) and after (T_2_) immersion in dist. H_2_O for 24 h, and continued for 5 weeks until constant thickness obtained and the thickness calculated using the following equation^27^:

2 $${\text{Thickness}}\;{\text{swelling }}\left( \% \right) = \left( {{\text{T2 }} - {\text{ T1}}} \right)/{\text{T1}} \times {1}00\% .$$

T_1_ = Thickness before soaking. T_2_ = Thickness after soaking.

The chemical resistance analysis was performed using ASTM D543 standard procedure, the test was performed on both treated and untreated composites. They were weighed (W_1_), immersed separately in 10% HCl, H_2_SO_4_, NaOH, KOH for 48 h, re-weighed after immersion (w_2_) and percentage gain/loss was evaluated using Eq. (1).

The density test was determined according to ASTM D792. The samples were cut into 2 × 4 mm in size. The mass was determined using weighing balance and the volume was measured using a micrometre screw gauge. The test was performed three times for each composite and the average value taken (Eq. 3)^28^.

3 $${\text{Density g}}/{\text{cm}}^{{3}} = \, \left( {\text{m}} \right)/\left( {\text{V}} \right)$$

m = Composite mass; V = Composite volume.

The tensile strength of the composites was performed using ASTM D638 Standard with Shimadzu (MODEL AG-1) machine. Tensile property was determined for seven different specimens of each sample composite and an average of three replicates of the tested specimens was presented. The method determines the tensile properties of the composite based on the stipulated conditions of fibre treatment, temperature and speed of the testing machine.

The flexural strength was determined using the Universal material Testing Machine (UTM), Shimadzu (Model AG-1) according to ASTM-D 790 at a cross head speed of 20 mm/min and a support of 51 mm. The samples were cut into rectangular specimens.

The hardness test was carried out using the hardness tester (Muver Franscisco, munoz Ireles, model 5019), using 30 × 30 × 3 mm dimension. The tests were performed across three sectional surfaces of each specimen of the composites.

Impact testing was carried out according to the standard specified by ISO 179 ASTM D-256 using the Ceast resil impactor testing machine P/N 6957 IZOD. The sample specimens were cut into 80 × 15 × 3 mm dimension.

The morphology of the composite samples were investigated using Pox: Phenom World scanning electron microscope (Model 800–07,334) with magnification range 500x, resolution 120 Å and acceleration of 15 kV^28^.

The FTIR analyses were determined with an FT-IR spectrometer (FTIR-Cary 630 from Agilent technologies and the spectra were recorded in the wavelength interval range of 4000–600 cm^−1^ with a resolution of 4 cm^−1^.

### The insect repellent property of the composites

The Insect Repellent Test was conducted according to American wood-preservers association (AWPA) standard^29^. A glass jar fitted with net cover was used, termites (30) were added to each glass jar containing 150 g fresh sand moistened with 30 cm^3^ distilled water, and the composite was then placed in the centre of each jar. The control jar was set up with a composite fabricated with no trace of *Canarium schweinfurthii* and termites (30). The test was performed in a dark environment. The repellent activity was observed and mortality rate was recorded. The same method was adopted for cockroaches.

## Results and discussion

### Physico-chemical analysis

The results obtained for all the physico-chemical analysis is presented in Table 2 and the figures are in the supplementary material. Moisture absorption of 0.288–0.42% was observed for the untreated fibre composites. Absorption up to 0.42% was due to hemicellulose and lignin contents responsible for the hydrophilic nature of the composites. Benzoylated (treated fibre) gave 0.18–0.085% due to presence of benzoyl. Control composite without fibre showed no water absorption due to absence of fibre and presence of LDPE (supplementary Fig. S6). The high fibre-matrix interaction resulted in a more hydrophobic composite and a decreased water absorption due to the benzoyl treatment. This result is in accordance with the findings of Rakesh et al.,^14^, Majid et al.,^30^ and Xue et al.,^18^ who reported the different chemical modifications on natural fibres for use in natural fibre-reinforced composites.

**Table 2:** Results obtained for the physico-chemical analysis.

	Control	10% fiber content		20% fiber content		30% fiber content	
		Treated	Untreated	Treated	Untreated	Treated	Untreated
Water absorption (wt.%)	0	0.085	0.288	0.139	0.3000	0.179	0.415
Density (g/cm^3^)	0.0099	0.0092	0.0097	0.0094	0.0096	0.0096	0.01
Tensile Strength (MPa)	33.0	28.73	17.78	31.90	25.24	35.08	27.78
Flexural strength (MPa)	138.9	436.5	406.6	444.0	411.0	456.3	426.4
Hardness (HV)	97	93	94	94	95	95	96
Impact load (J/m)	850	781	820	744	801	730	779

The result obtained for the thickness swelling of the modified composites showed that the composites did not swell when kept for 5 weeks in distilled water, this is attributed to the high content (100 g) of waste LDPE in the composites. Similar result was revealed by Gulitah and Liew^31^.

The results of the density test (supplementary Fig. S7) showed a low density for all of the composites tested. The highest value obtained was 0.01 g/cm^3^ (for 30% untreated fibre composite), the weight increases with increase in fibre content attributing to the presence of lignin and hemicellulose (i.e. moisture absorption increases density). Benzoylated fibre composites showed a decrease in density due to partial removal of lignin and hemicellulose^32^ in the alkali pre-treated fibre with subsequent benzoyl group introduction, this is in accordance with^33,34^. The benzoylation treatments reduce the hydrophilicity of the fibre which consequently improves the fibre adhesion to the hydrophobic matrix in the composites. Highly hydrophobic phenyl group moieties are introduced into the fibre to reduce its hydrophilicity. This enhances the fibre adhesion with the highly hydrophobic LDPE matrix and improves fiber matrix interaction.

The tensile strength analysis shows increases with increase in fibre content (supplementary Fig. S8), all the composites including the control showed high tensile strength linked to the strong adhesion between the matrix and binder. The control composite has a high tensile strength of ~ 33 MPa compared to untreated tiger nut fibre composites (i.e. 17.78, 25.24 and 27.78 MPa). This is because of weak compatibility between the fibre and the matrix due to hemicellulose, lignin and pectin present. The treated tiger nut fibre composites showed improved tensile strengths beyond the control (35.08 MPa). Therefore, benzoylation increases the hydrophobicity of the fibre, resulting in high fibre-matrix interaction and mechanical properties, as previously reported in literatures^18,20,30,34^.

The flexural strength analysis also increases with increase in fibre content and fibre treatment (supplementary Fig. S9). The control with no fibre had the lowest bending stress of 138.9 MPa, compared to untreated composite 426.4 MPa. The benzoylated fibre composites have higher bending stresses, 456.3 MPa compared to the untreated composite, attributing to partial removal of hemicellulose and lignin. Generally all the composites have high bending stress due to the polymers properties incorporated into the composites through the waste LDPE. The results obtained are in accordance with the Favaro et al.,^28^ with findings showing that flexural strength was found to increase after chemical modification.

The indentations of the composites increased with increasing fibre content but decreased with fibre modification (supplementary Fig. S10). The control composite showed a high hardness testing value (97 HV) indicating no fibre-matrix interaction due to the absence of fibre. All untreated fibre composites were found to have slightly higher indentations compared to the treated ones. This indicates that the treated composites are harder due good fibre matrix interaction as a result of the benzoylation. The hardness test result obtained is related to the work of Onuegbu^34^.

The impact tests shows that the control composite has the highest impact energy of 850 J/m due to the lack of fibre matrix adhesion (supplementary Fig. S11). The untreated fibre composites show high impact energies of 820, 801 and 779 J/m compared to benzoyl chloride treated fibre composites 781, 744 and 730 J/m respectively. This can be related to the very good fibre-matrix adhesion in the treated fibre composites since low impact load implies an increase in fibre matrix adhesion. The result is similar to that of Jang and Lee works^35^.

**Figure 2:**
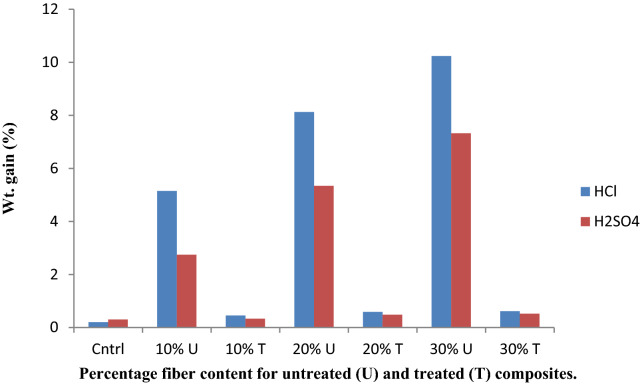
Acid chemical resistance test of the composite.

**Figure 3:**
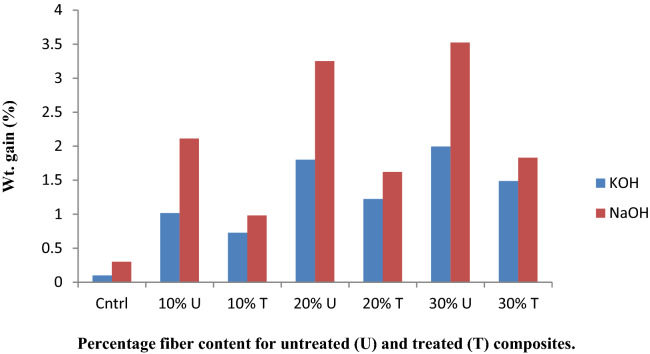
Base chemical resistance test of the composite.

The chemical resistance test results obtained are shown in Figs. 2 and 3, respectively. The weight gain observed by the composites, for all the chemical reagents used, indicated that the composites were swollen as a result of the composites interaction with the chemical reagents. This results in gel formation rather than dissolving in the chemical reagents. The weight gain shown by the treated composites was low compared to the untreated composites, due to close packing of the matrices and reinforcement. Meanwhile, higher weight gains are observed with acid treatment than with base. This shows that the composites react more in acidic medium. The results are in accordance with the work of Yakasai^32^ and Venkatesha et al.,^33^.

### Scanning electron microscopy (SEM)

**Figure 4:**
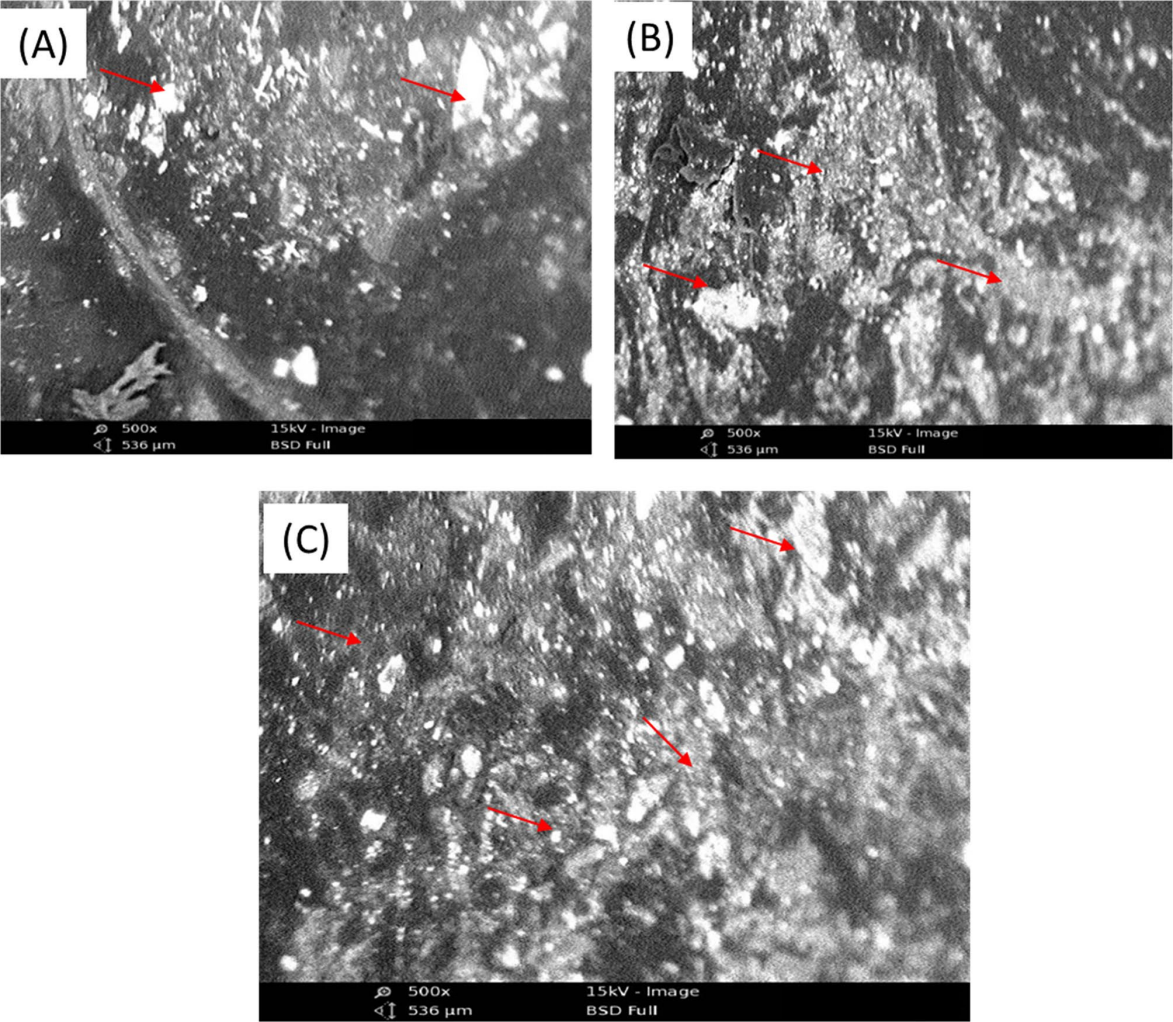
SEM Micrograph of (**A**) Composite with no particulate fibre, (**B**) composite with untreated tiger nut particulate fibre, (**C**) composite with treated tiger nut particulate fibre.

Figure 4A is the SEM image of the control composite, which shows visible white spots, indicating the interaction between only the matrix and binder. Figure 4B shows the SEM image of untreated tiger nut fibre composite with increased visible white spots, which appear chunky and agglomerated due to the interactions between the matrix, binder and fibre, wax, and surface impurities from the untreated fibre, which provide a surface protective layer. The surface roughness of the tiger nut fibre could be an indication of lignin presence. The effect of benzoylation was shown in Fig. 4C where the visible white spots increased more and are widely dispersed on the surface. This could be an indication of increasing fibre-matrix interaction due to benzoylation resulting in better adhesion. The treatment provides direct bonding between the matrix and cellulose. It can be clearly seen that the morphology of the treated tiger nut fibre resulted in separation of the microfibrillar because of the delignification. This result is similar to the findings of Rakesh et al., Salisu et al., Yakasai, and Webo et al.,^14,27,32,36^ on SEM analyses of alkalized, silylated and benzoylated natural fibres.

### FT-IR-spectroscopy

The FTIR spectra are shown (supplementary Fig. S12–S18) for untreated and benzoyl chloride treated fibre composites respectively, with their assignments shown in supplementary table S3-S9. Untreated fibre composites showed bands at 1380 cm^−1^ and 1469 cm^−1^corresponding to C–H bending of alkyl group. An important modification was observed at 1722 cm^−1^ indicating C=O for stretching of the benzoyl carbonyl group from the benzoylation treatment. No band was observed between 3000 and 4000 cm^−1^ in both untreated and benzoyl chloride treated composite, but the region of the hydroxyl group in the untreated composite is broader, indicating the hydrophilic nature compared to that of the treated. This clearly shows the hydrophobic nature of the composites, in agreement with water absorption and thickness swelling results. The general hydrophobic nature of the composites is attributed to the incorporation of the waste LDPE and the hydrophobicity of the composite increases with the benzoyl chloride treatment.

### The insect repellent property of the composites

**Figure 5:**
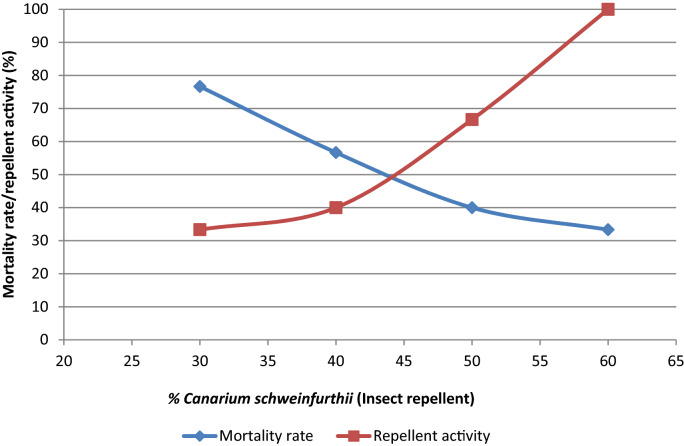
Mortality rate/repellent activity of termites towards the composites.

**Figure 6:**
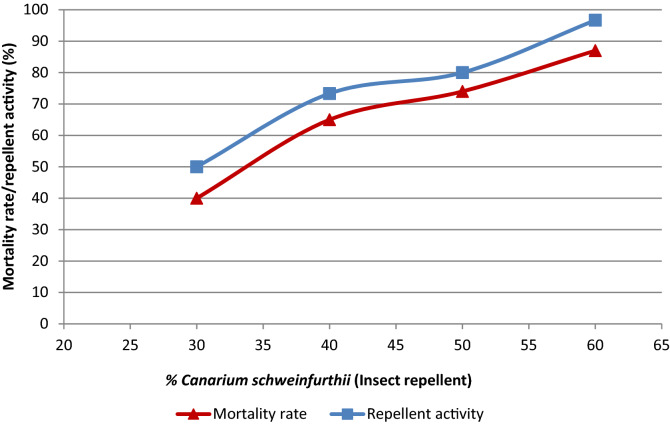
Mortality rate/repellent activity of cockroach towards the composites.

The result of the repellent activity and mortality rate of termites and cockroaches are shown in Figs. 5 and 6, respectively. The highest repellent activity was observed with the composite with 60% binder (*Canarium Schweinfurthii*). This indicates that the increase in concentration of the binder increases the repellent activity. The highest mortality rate of termites was observed on the composite with 30% *Canarium Schweinfurthii* gum (binder); this is due to the low concentration of *Canarium Schweinfurthii* gum (binder) that allows them to feed on it and die. The cockroaches showed a reverse case, both the repellent activity and mortality rate increased with increase in the concentration of binder. The highest repellent and mortality rate was observed on the composite with 60% *Canarium Schweinfurthii* gum (binder). The repellent activity is higher than the mortality rate because the smell alone has a great effect on the cockroach. This finding agrees with the studies conducted by Wannang et al.,^37^ who reported that when snakes perceived the smell of burning exudate of *canarium schweinfurthii* and the scent transferred to their jacobson’s organ (taste and scent receptor), they may experience signs of toxicities, and be repelled. So also, this may apply to the termites and cockroaches, since once they come in contact with the composite and the scent is transferred to their organ, they may get irritated, repelled and even die. Also, Wong and Grace^28^ evaluated Formosan subterranean termite resistance of borate-treated rubber-wood chipboard using both no-choice and two choice methods.

## Conclusion

The insect repellent hybrid composites were successfully fabricated and characterised based on their physico-chemical, physico-mechanical, morphological, chemical interaction and insect repellent properties. The treated fibre composites showed remarkable improvement in water absorption, thickness swelling, chemical resistance, density, and physico-mechanical properties compared to the untreated fibre composites. All the composites were hydrophobic due to the waste LDPE incorporated with increase in the hydrophobicity of treated fibre composite due to good fibre-matrix interaction. FTIR analysis of both fibres and composites shows the effectiveness of benzoylation treatments. The alkyd resin from the free fatty acid had an excellent performance**.** The NaOH modification of tiger nut fibre activated the hydroxyl groups, aiding the benzoyl chloride reaction with the fibre, resulting in an improved hydrophobicity of the fibre, as well as improvement in the fibre-matrix interaction. The insect repellent property of the composite was successful, as evident from the observed repellent activity and mortality rate of the insects tested. In view of the composite physico-chemical, physico-mechanical, morphological, chemical interaction and insect repellent properties, the composite has a promising application in furniture making and ceiling panels to repel insects (termites and cockroaches).

## Supplementary Information

Supplementary Information.

## Data Availability

The data that support the findings of this work are available upon reasonable request from the corresponding author.
