# Supplementary material for: Fabrication of benzoyl chloride treated tiger-nut fiber reinforced insect repellent hybrid composite
Source: Sci Rep. 2022 May 25;12:8797. doi: 10.1038/s41598-022-12876-0 (PMC9132963; doi:10.1038/s41598-022-12876-0)
Supplement: Supplementary file 1 — Supplementary Information. [file 41598_2022_12876_MOESM1_ESM.docx]

**Supplementary Information**

**Fabrication of benzoyl chloride treated tiger-nut fiber reinforced insect repellent hybrid composite**

Hajara Babayo ^a, b^, Haruna Musa ^a^, Mustapha D. Garba ^a,^ *

^a^ Department of Pure and Industrial chemistry, Bayero University Kano, Nigeria P.M.B 3011

^b^ Fibre and Polymer Science Program, North Carolina State University Raleigh, NC, 27606, USA

e-mail: [m.d.garba.11@gmail.com](mailto:m.d.garba.11@gmail.com)


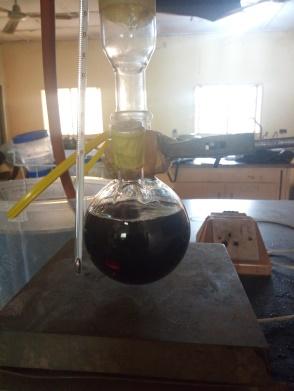

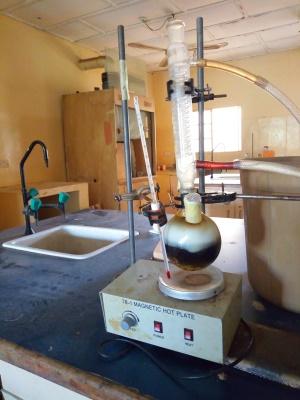


Stage 1: Start of the reaction Stage 2: Formation of homogenous solution


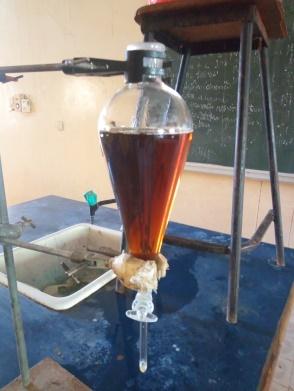

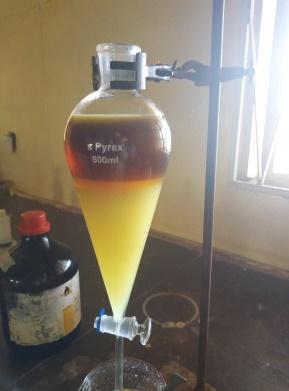


Stage 3: Dissolution of soap layer Stage 4: Formation of FFA at the top layer

**Fig. S1:** Extraction of free fatty acid; Stage 1 is the saponification of castor oil with a solution of sodium hydroxide, (1:1) ethanol and distilled-water. Stage 2 is the refluxing stage where a homogenous mixture is obtained. Stage 3 is the phase separation stage where the soap was obtained. At the final stage the soap was reacted with sulfuric acid to obtain free fatty acid.


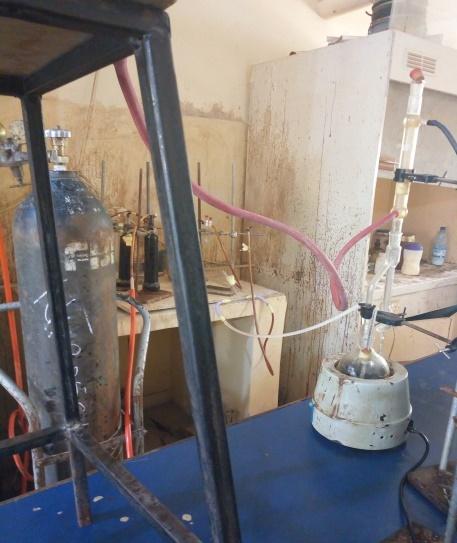

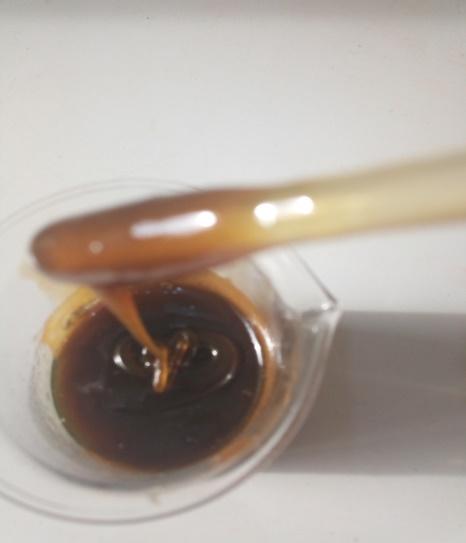


a) Set up for the preparation of alkyd resin b) Prepared alkyd resin.

**Fig S2:** Images for alkyd resin preparations


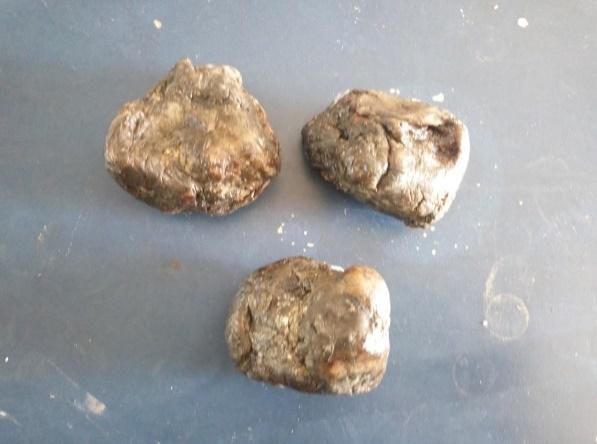

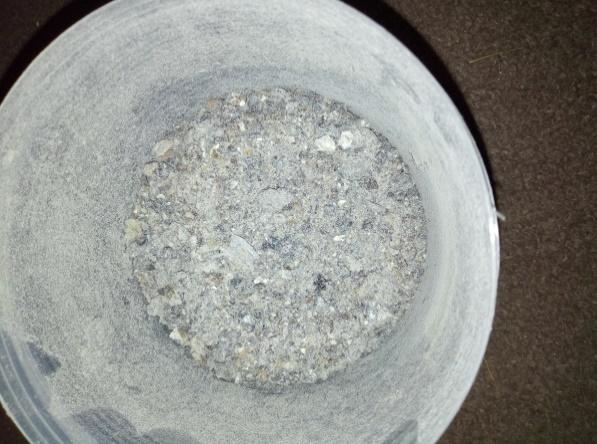


a) Raw *Canarium schweinfurthii* gum. b) Dried powder of *Canarium schweinfurthii* gum.


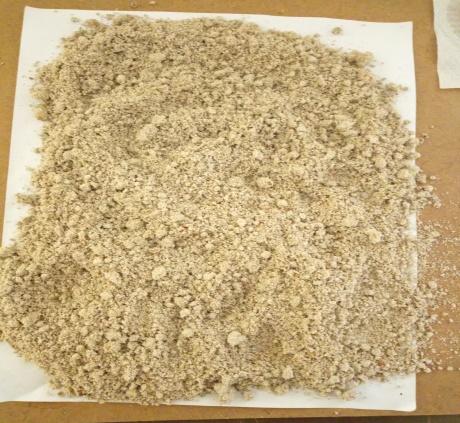

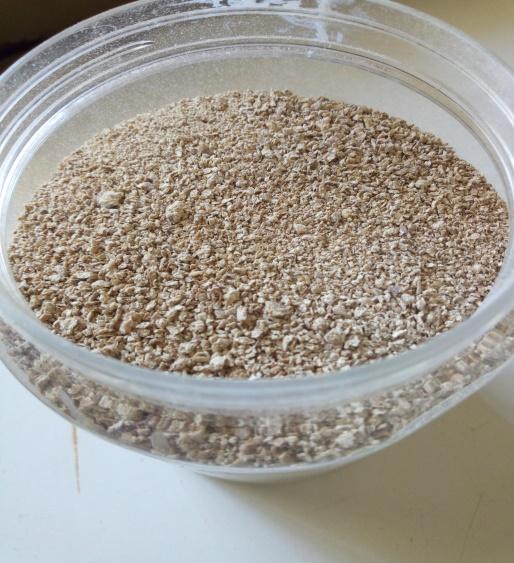


c) Dried untreated tiger nut fibre. d) Benzoyl chloride treated tiger nut fibre.

**Fig S3:** Tiger nut and *Canarium schweinfurthii* gum treatments


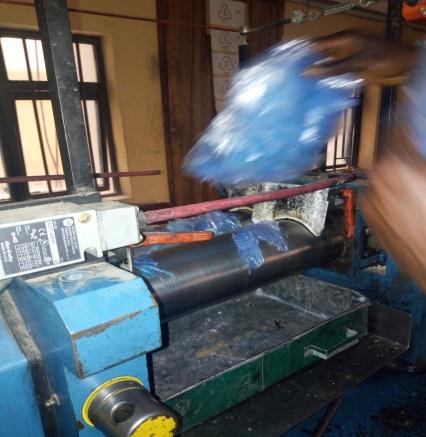

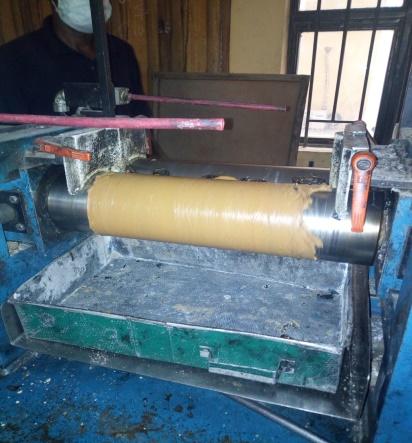


Step 1: Melting of LDPE

Step 2: Mixing of all the materials


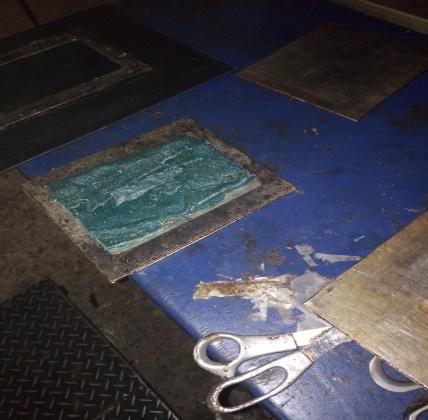

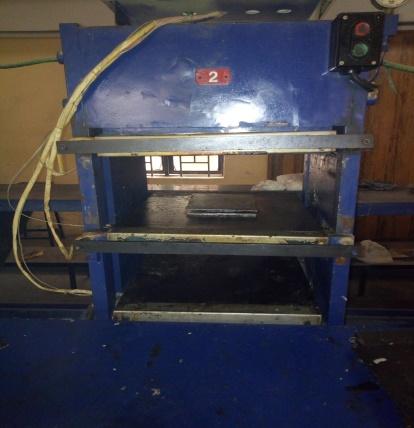


Step 4: Compression Moulding

Step 3: Semi-finished composite (Sheet Moulding Compound)


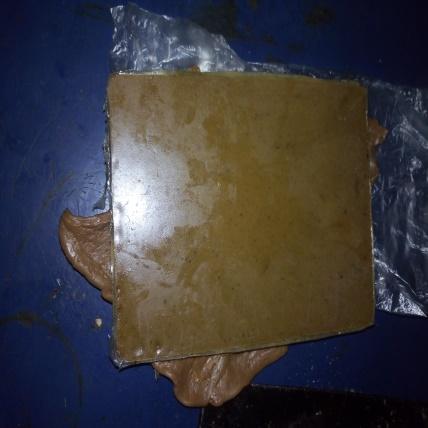


Stage 5: Molded Composite

**Fig. S4:** Process of composite fabrication; step 1 is where the waste LDPE was melted, all components were mixed in step 2, the semi-finished composite was prepared in a mould in the 3^rd^ step and compressed in the 4rd step, the final step is the moulded composite obtained.


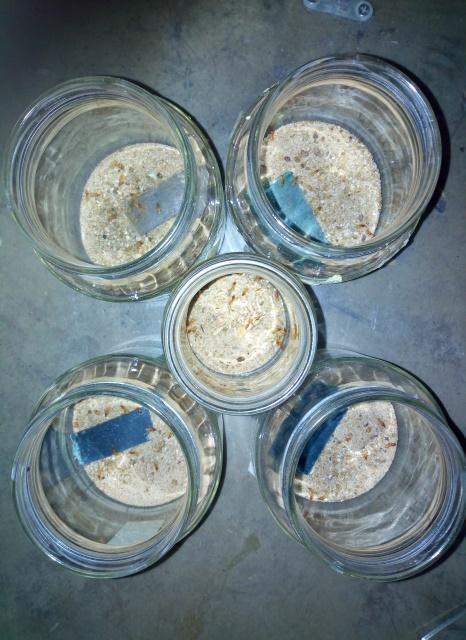

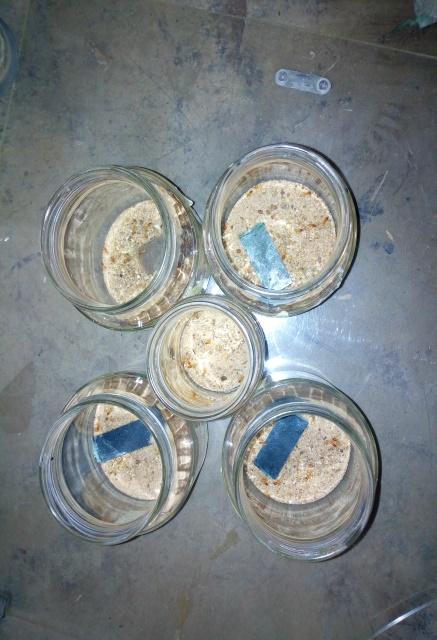


a) Termite’s repellent activity/mortality rate


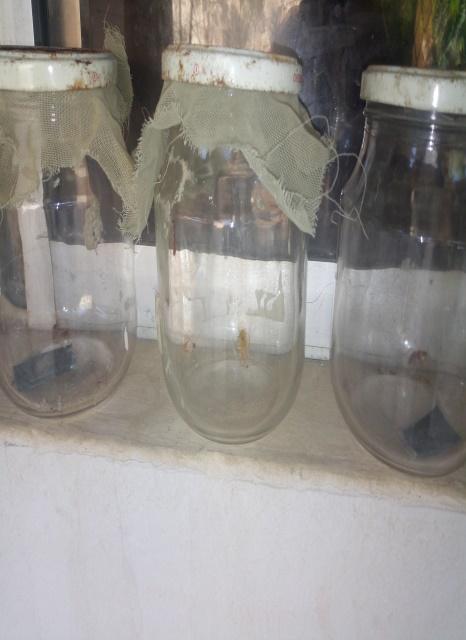


b) Cockroach repellent activity/ mortality rate

**Fig. S5:** Insect repellent test


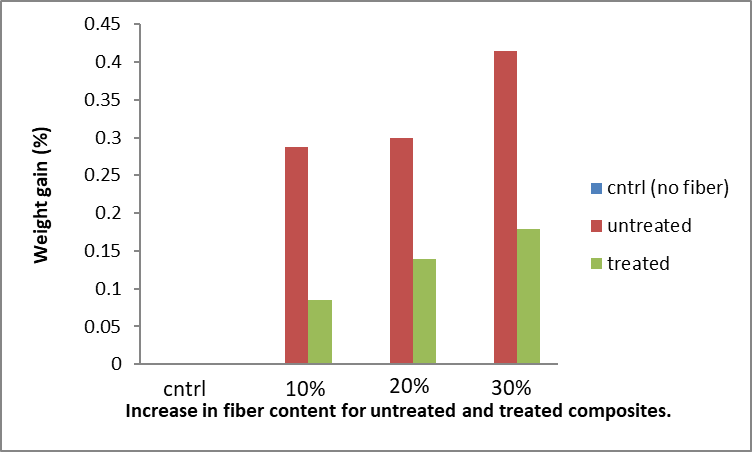


Fig. S6: Water absorption of composites


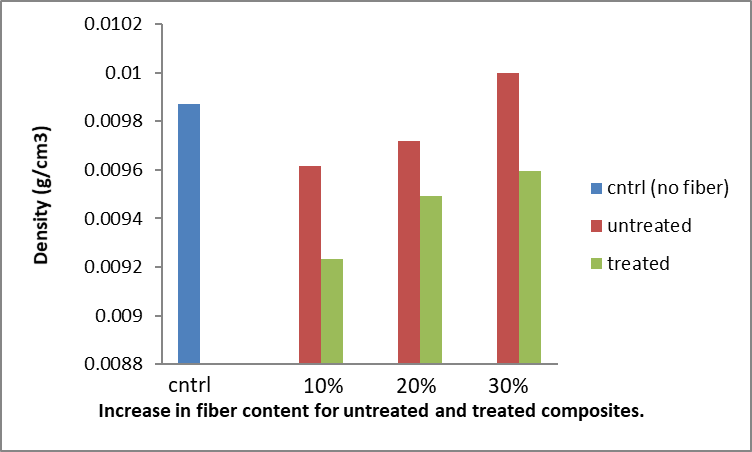


Fig.S7: Density of the composites


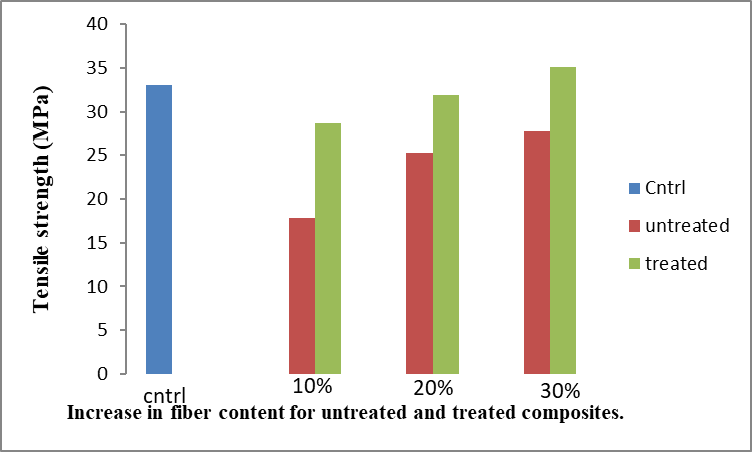


Fig. S8: Tensile Strength of Composites


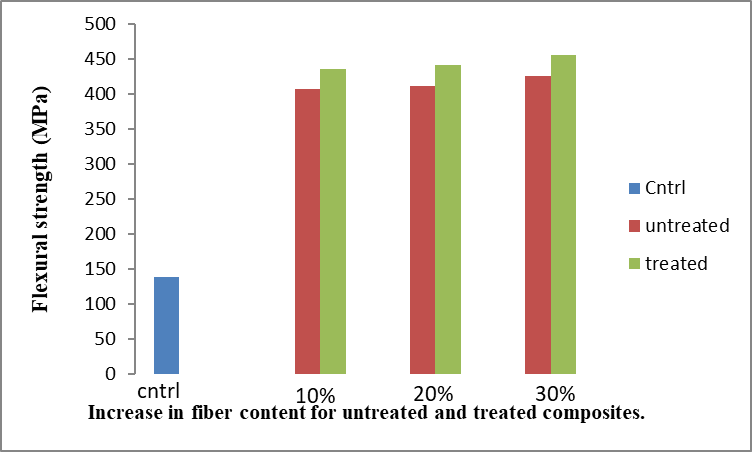


Fig. S9: Flexural strength of composites


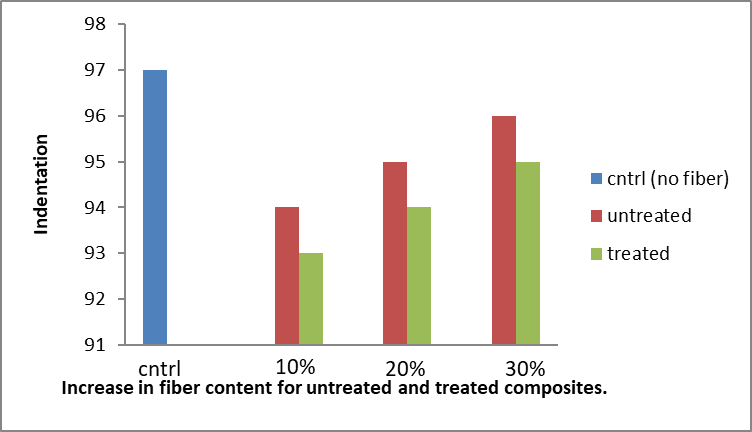


Fig. S10: Hardness of the composites


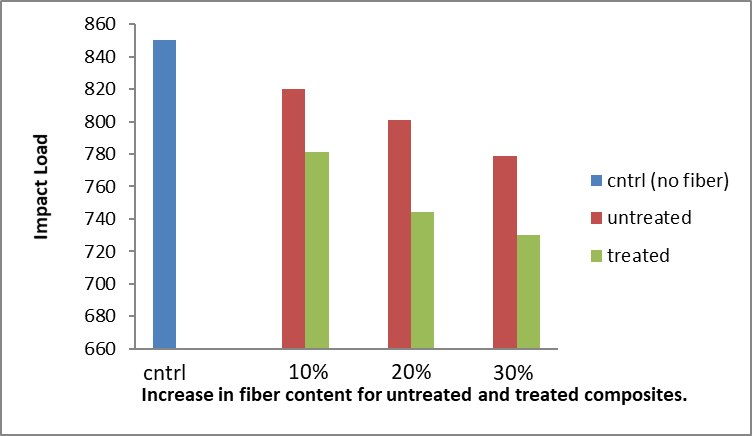


Fig. S11: Impact load of composites


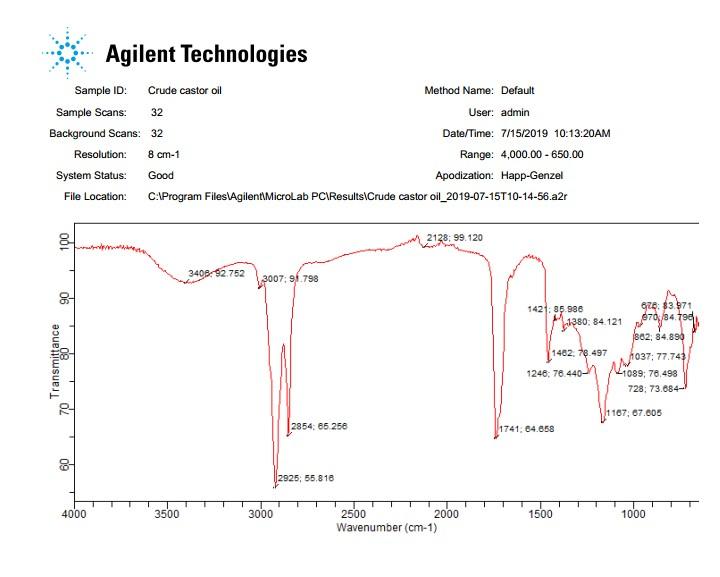


**Fig. S12:** FTIR of Crude castor oil


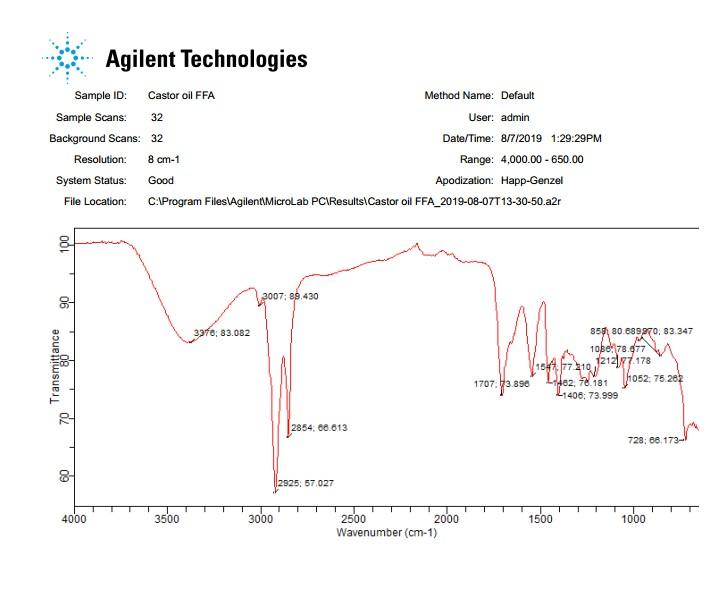


**Fig. S13:** FTIR of castor oil FFA


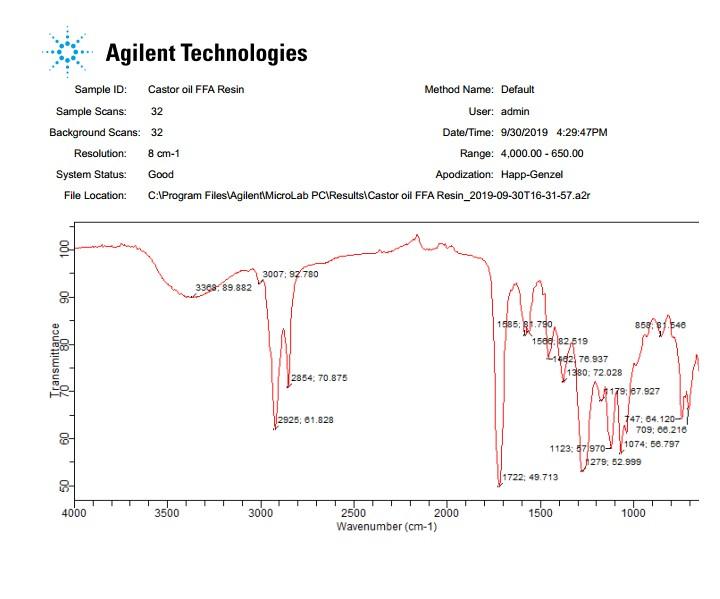
**Fig. S14:** FTIR of castor oil FFA Resin


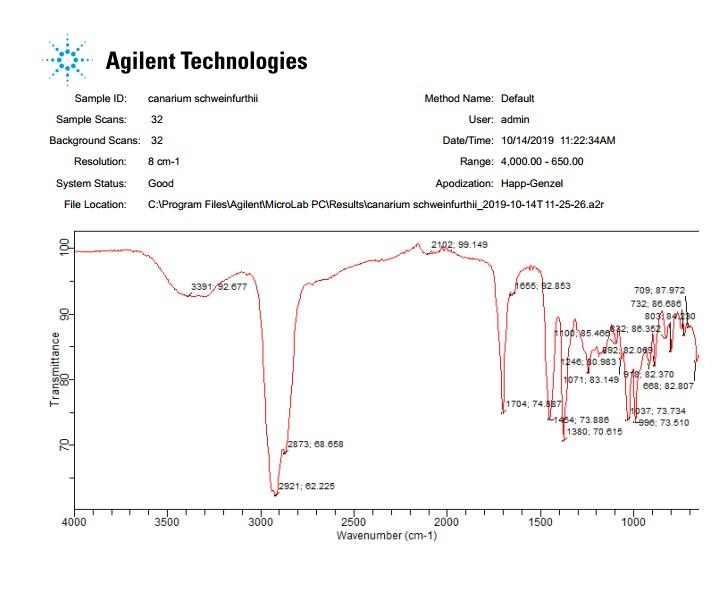
**Fig. S15:** FTIR of canarium schweinfurthii gum resin


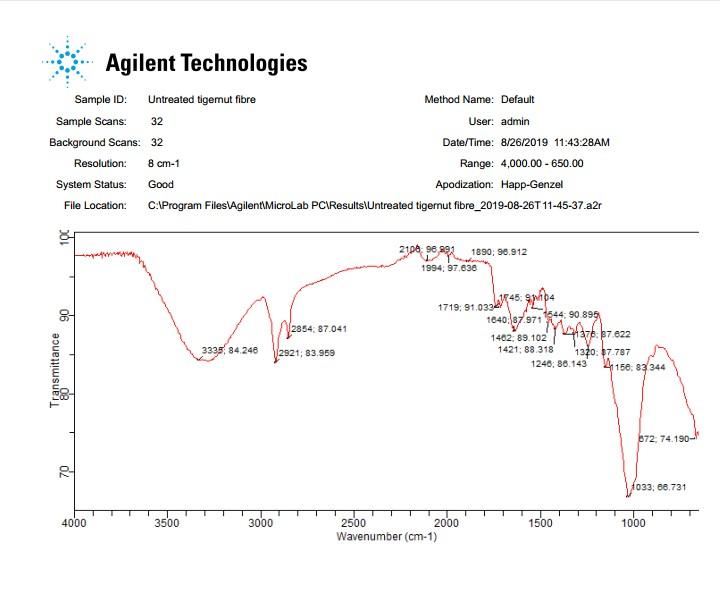


**Fig. S16:** FTIR of untreated tiger nut fibre


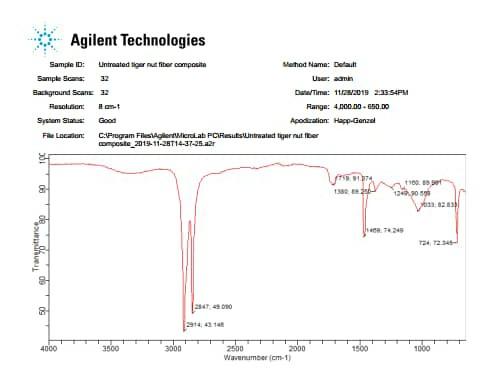


**Fig. S17**: FTIR of Benzoyl Chloride Treated Tiger Nut Fibre


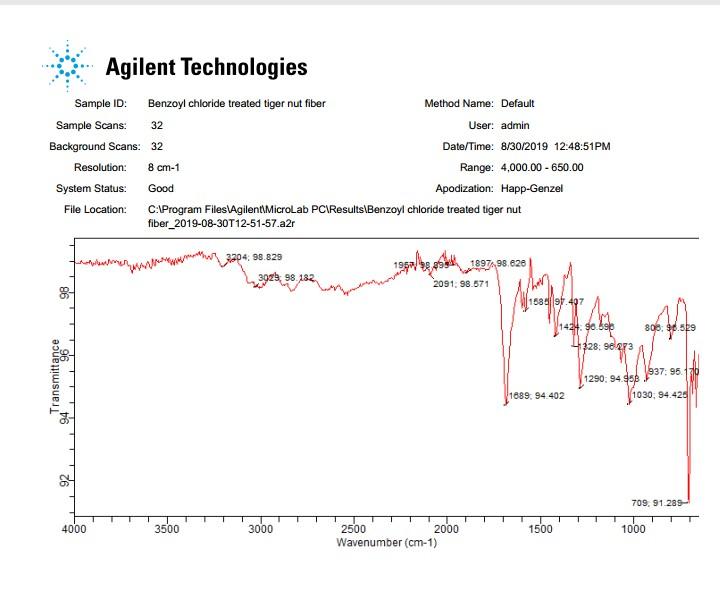
**Fig. S18**: FTIR of untreated Tiger Nut Fibre composite

**
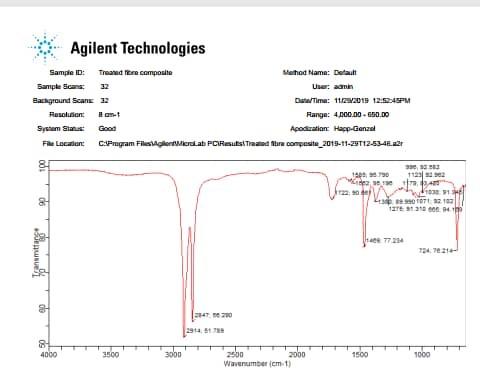
** **Fig. S18:** FTIR of Benzoyl Chloride Treated Tiger Nut Fibre composite

**Table S1:** Physicochemical properties of the crude castor oil

| Characteristics | Unit | Crude castor oil | Methods Used | Instrument |
| --- | --- | --- | --- | --- |
| Acid value | mg NaOH/g | 36.91 | BinQuader *et al*., (2018) |  |
| Specific density at 30^0^C | g/cm^3^ | 0.956 | BinQuader *et al*., (2018) |  |
| Saponification value | mg/g | 216 | Jibrin *et al*., (2018) |  |
| Iodine value, Hanus | gI_2_/g | 179.8 | BinQuader *et al*., (2018) |  |
| Viscosity | MPas | 218.3 |  | Clandom viscometer, model VT-03 |
| pH |  | 4.71 | BinQuader *et al*., (2018) |  |
| Moisture Content | % | 1.33 | Abdulkadir and Jimoh., (2013) | Denver moisture analyzer (model IR-35) |
| FTIR | cm^-1^ |  |  | Car 630;Agilent Technology |

**Table S2:** Physical and Chemical Properties of Alkyd Resin.

| **Properties** | **Specific value** | **Alkyd resin from castor oil** |
| --- | --- | --- |
| Colour |  | Brown |
| Viscosity (absolute) | MPas | 419.63 |
| Solubility in; | Xylene  Acetone  Toluene  Ethanol  Cyclohexanol | Completely soluble  Completely soluble  Completely soluble  Insoluble  Limited solubility |
| FTIR | cm^-1^ |  |

**Table S3:** FTIR absorption bands of the crude castor oil

| Experimental frequency (cm^-1^) | Literature frequency (cm^-1^) | Remark |
| --- | --- | --- |
| 3007.91 | Near 3030 | =C-H |
| 3406.92 | 3570-3200 | O-H stretch |
| 1167.67 | Near 1100 | C-O stretch |
| 2925.55  2854.65 | 2926-2850 | C-H |
| 1462.78  1421.85 | 1485-1415 | C-H |
| 1741.64 | 1750-1735 | C=O |
| 1246.76  1167.67  1089.76 | 1245-1089 | C-O |

**Table S4:** FTIR absorption bands of the castor oil FFA

| Experimental frequency (cm^-1^) | Literature frequency (cm^-1^) | | Remark |
| --- | --- | --- | --- |
| 3007.91 | 3010 – 3030 | =C-H Olefinic C-H stretching, this is a clear indication of the unsaturated fatty acids. | |
| 3378.83 | 3570-3200 | O-H Broad peak around 3400 cm^-1^ corresponds to the hydroxyl group of high concentrated carboxylic acids. | |
| 1707.73 | 1710 | C=O carbonyl stretching of carboxylic acids | |
| 2925.55  2854.65 | 2926-2850 | C-H | |
| 1482.78 | 1485-1440 | C=C | |
| 1406.73 | 1410 | COO^-^ Cis group | |
| 1212.77  1086.78  1052.75 | 1245-1030 | C-O | |
| 970.83 | 936-980 | Dimer of carboxylic acid | |
| 728.66 |  | The rocking of multiple (>4) methylene groups | |

**Table S5:** FTIR absorption bands of the castor oil FFA Resin (Hlaing and Oo., 2008: Sharif 2015)

| Experimental frequency (cm^-1^) | Literature frequency (cm^-1^) | Remark |
| --- | --- | --- |
| 3368.89 | 3200-2700 | O-H stretching |
| 3007.82 | 3030-3000 | C-H olefinic stretching due to fatty acid. |
| 2925.61  2854.70 | 2926-2850 | C-H |
| 1722.49 | 1730-1730 | C=O due to ester |
| 1585.81  1566.82 | 1600-1550 | Aromatic C=O stretching of the phthalate system. |
| 1462.76 | 1480-1460 | -CH_2_ bending vibration. |
| 1380.72 | 1343-1385 | -CH_3_ bending |
| 1279.52  1179.67  1123.57 |  | C-O stretching |
| 1074.56 | 1074-1000 | -C-O stretching of ether. |
| 747.64 | 750-730 | Rocking of multiple (>4) methylene groups of the fatty acids. |

**Table S6:** FTIR absorption bands of *Canarium Schweinfurthii*

| Experimental frequency (cm^-1^) | Literature frequency (cm-^1^) | Remark |
| --- | --- | --- |
| 3391.92 | 3400-3200 | O-H stretching |
| 2873.68  2921.62 | 2926-2880 | C-H |
| 1704.74 | 1725-1705 | C=O |
| 1454.73 | 1485-1440 | C=C stretching |
| 1380.70 | 1380-1370 | -C-H bending bend. |
| 1246.80  1100.85  1071.83 | 1245-1030  1150-1050 | C-H bends.  C-O stretching |
| 918.82  996.73 | 900-600 | C-H out of plane |
| 892.82 | 900-600 | C-H out of plane |
| 709.87 | 900-600 | Rocking of multiple (<4) methylene group |
| 668.82 | 900-600 | C-H out of plane |

**Table S7:** FTIR absorption bands of the untreated tiger nut particulate fibre

| Experimental frequency (cm-^1^) | Literature frequency (cm-^1^) | Remark |
| --- | --- | --- |
| 3335.84 | 3570-3200 | O-H Broad peak around 3400 cm^-1^corresponds to the hydroxyl group. |
| 2921.83  2854.87 | 2926-2850 | C-H |
| 1719.91  1745.91 | 1760-1720 | C=O carbonyl of lignin in cellulose |
| 1462.89 | 1485-1440 | C=C |
| 1640.87 | 1680-1640 | C=O |
| 1376.87 | Near 1380 | C-H hemicellulose |
| 1156.83 | 1200-1100 | C-O |
| 672.74 | 700-670 | C-H |

**Table S8:** FTIR absorption bands of Benzoyl Chloride Treated Tiger Nut Particulate Fibre

| Experimental frequency (cm-^1^) | Literature frequency (cm-^1^) | Remark |
| --- | --- | --- |
| 3204.98 | 3200-2700 | O-H weak peak corresponds to the hydroxyl group present in cellulose. |
| 3029.98 | 3070-3020 | =C-H stretching of aromatic due to phenyl nucleus |
| 1689.94 | 1770-1640 | C=O due to benzoyl carbonyl. |
| 1424.96 | 1424-1415 | Conjugated C-H bending vibrations. |
| 1328.96 | 1343-1319 | C-H in plane bending of hemicellulose. |
| 1290.94 | 1290-1266 | C-O syringyl ring breathing and C-O stretching in lignin and Xylan. |
| 1030.94 | 1043-1028 | C-O, C=C, symmetric glycosidic stretching. |
| 937.95 | 980-936 | Dimer of carboxylic acid |
| 808.96 | 860-800 | C-H |

**Table S9:** FTIR absorption bands of untreated and benzoyl chloride treated tiger nut particulate fibre composites

| Experimental frequency  (cm-^1^)Untreated Composite | Experimental frequency (cm-^1^)Treated Composite | Literature frequency (cm-^1^) | Remark |
| --- | --- | --- | --- |
| 2847.49 | 2847.56  2914.51 | 2926-2850 | C-H |
| _ | 1722.90 | 1725-1700 | C=O due to benzoyl carbonyl. |
| 1469.74 | 1469.77 | 1470-1415 | C-H symmetrical bending vibration. |
| 1380.89 | 1380.89 | 1380-1319 | C-H in plane bending of hemicellulose. |

**Table S10:** Analysis of the composites

| Analysis | Instruments used | Unit | Composites | |
| --- | --- | --- | --- | --- |
|  |  |  | Untreated | Treated |
| Water absorption ASTM D-570 |  | Wt% | 0.288 | 0.085 |
| Thickness swelling ASTM D-570 |  | Wt% | No effect | No effect |
| Chemical resistance (Acids) ASTM D543 |  | Wt% | 2.734 (H_2_SO_4_)  5.146 (HCL) | 0.331  0.450 |
| Chemical resistance (Bases) ASTM D543 |  | Wt% | 1.016 (KOH)  2.113 (NaOH) | 0.728  0.982 |
| Density ASTM D2395-17 |  | g/cm^3^ | 0.0096 | 0.0092 |
| Tensile strength ASTM D-638 | Tensile test (Universal material testing machine, Shimadzu, model AG-1) | Mpa | 27.78 | 35.08 |
| Flexural strength ASTM D-792 | flexural test (Universal material testing machine, Shimadzu, model AG-1) | Mpa | 426.4 | 456.3 |
| Hardness | hardness tester (Muver Franscisco, munoz Ireles, model 5019) | HV | 95 | 93 |
| Impact ASTM D-256 | Impact tester (Ceast Resil impactor, model P/N 6957 IZOD) | J/m | 779 | 730 |
| SEM (Favaro *et al*., 2010) | Scanning Electron Microscope (Prox: Phenom World 800-07334) |  |  |  |
| FTIR | Fourier Transform Infrared spectrophotometer (Car 630; Agilent Technology) | cm^-1^ |  |  |
